# Identification and Characterization of Novel Variations in Platelet G-Protein Coupled Receptor (GPCR) Genes in Patients Historically Diagnosed with Type 1 von Willebrand Disease

**DOI:** 10.1371/journal.pone.0143913

**Published:** 2015-12-02

**Authors:** Jacqueline Stockley, Shaista P. Nisar, Vincenzo C. Leo, Essa Sabi, Margaret R. Cunningham, Jeroen C. Eikenboom, Stefan Lethagen, Reinhard Schneppenheim, Anne C. Goodeve, Steve P. Watson, Stuart J. Mundell, Martina E. Daly, GAPP study in collaboration with the MCMDM-1VWD study group

**Affiliations:** 1 Department of Cardiovascular Science, University of Sheffield, Sheffield, United Kingdom; 2 School of Physiology and Pharmacology, University of Bristol, Bristol, United Kingdom; 3 Department of Thrombosis and Hemostasis, Leiden University Medical Center, Leiden, The Netherlands; 4 National Haemophilia Center, University Hospital, Rigshospitalet, Copenhagen, Denmark; 5 Department of Pediatric Hematology and Oncology, University Medical Center Hamburg-Eppendorf, Hamburg, Germany; 6 Centre for Cardiovascular Sciences, College of Medical and Dental Sciences, University of Birmingham, Birmingham, United Kingdom; University of Pennsylvania School of Medicine, UNITED STATES

## Abstract

The clinical expression of type 1 von Willebrand disease may be modified by co-inheritance of other mild bleeding diatheses. We previously showed that mutations in the platelet P2Y_12_ ADP receptor gene (*P2RY12*) could contribute to the bleeding phenotype in patients with type 1 von Willebrand disease. Here we investigated whether variations in platelet G protein-coupled receptor genes other than *P2RY12* also contributed to the bleeding phenotype. Platelet G protein-coupled receptor genes *P2RY1*, *F2R*, *F2RL3*, *TBXA2R* and *PTGIR* were sequenced in 146 index cases with type 1 von Willebrand disease and the potential effects of identified single nucleotide variations were assessed using *in silico* methods and heterologous expression analysis. Seven heterozygous single nucleotide variations were identified in 8 index cases. Two single nucleotide variations were detected in *F2R*; a novel c.-67G>C transversion which reduced *F2R* transcriptional activity and a rare c.1063C>T transition predicting a p.L355F substitution which did not interfere with PAR1 expression or signalling. Two synonymous single nucleotide variations were identified in *F2RL3* (c.402C>G, p.A134 =; c.1029 G>C p.V343 =), both of which introduced less commonly used codons and were predicted to be deleterious, though neither of them affected PAR4 receptor expression. A third single nucleotide variation in *F2RL3* (c.65 C>A; p.T22N) was co-inherited with a synonymous single nucleotide variation in *TBXA2R* (c.6680 C>T, p.S218 =). Expression and signalling of the p.T22N PAR4 variant was similar to wild-type, while the *TBXA2R* variation introduced a cryptic splice site that was predicted to cause premature termination of protein translation. The enrichment of single nucleotide variations in G protein-coupled receptor genes among type 1 von Willebrand disease patients supports the view of type 1 von Willebrand disease as a polygenic disorder.

## Introduction

Type 1 von Willebrand disease is a highly heterogeneous bleeding disorder that is characterised by a partial quantitative deficiency of functionally normal von Willebrand factor (VWF) [1]. Its diagnosis is complicated by the incomplete penetrance of the disease and the wide variability in plasma VWF levels which are influenced by environmental (e.g. age, stress, exercise) and genetic factors (e.g. ABO blood group) [1]. While the pathogenetic mechanisms underlying the disease remain to be fully resolved, data from molecular epidemiological studies indicate that mutations in the VWF gene (*VWF)* are present in approximately 65% of index cases diagnosed with the disorder [2–4]. The majority of *VWF* sequence variations predict missense substitutions in VWF and the likelihood of identifying a variation is greater in those patients having more severe deficiency of the protein (3).

The search for genetic factors that may explain the bleeding tendency in the 35% of patients with no recognisable *VWF* mutations has focused mainly on the identification of modifier genes that have a role in influencing plasma VWF levels. Thus, in addition to the *ABO* locus which is an established contributor to variation in plasma VWF levels, genome wide association studies have identified several other genes encoding proteins that have roles in trafficking and clearance which have been shown to influence VWF levels [5,6]. The co-existence of other mild bleeding diatheses might also modify the expression of the bleeding tendency in patients diagnosed with type 1 VWD. Given the role of VWF in primary haemostasis, and the clinical similarities of patients with type 1 VWD and platelet bleeding disorders, the bleeding tendency in patients with type 1 VWD may be influenced by variation in the genes encoding the receptors and signalling proteins that mediate platelet adhesion and aggregation. Indeed, we have previously identified two mutations in the platelet P2Y_12_ ADP receptor gene (*P2RY12*) in patients with type 1 VWD who were recruited through the European *Molecular and Clinical Markers for the Diagnosis and Management of type 1 von Willebrand Disease* (MCMDM-1VWD) study and showed that they could contribute to the bleeding phenotype in these patients [7,8].

P2Y_12_ is one of several GPCRs expressed on the cell surface of platelets which, when activated, generate stimulatory or inhibitory signals that serve to both amplify and limit platelet recruitment and aggregation at sites of vessel injury. Platelet activation through GPCRs is mediated primarily via ADP, which, in addition to P2Y_12_, elicits its response through the P2Y_1_ ADP receptor; thromboxane A_2_ (TxA_2_), which elicits its response through the thromboxane receptor TP; and thrombin, which activates platelets through protease-activated receptors-1 and -4 (PAR1 and PAR4) [9]. The receptors P2Y_1_, TP, PAR1 and PAR4 are coupled via Gq to phospholipase Cβ2 (PLCβ2), activation of which results in an increase in cytosolic Ca^2+^ levels, leading to activation of phospholipase A_2_ (PLA_2_) and generation of TxA_2_ which, when released, activates additional platelets though TP [9,10]. Interaction of TP with TxA_2_, and of PAR1 and PAR4 with thrombin, also leads, via G_13_, to activation of Rho kinase and the cytoskeletal responses resulting in platelet shape change [11]. In contrast to the stimulatory effects of thrombin, ADP and TxA_2_, the major endothelium-derived inhibitor of platelet activation, prostacyclin (PGI_2_), mediates its protective effects through the platelet G_s_-coupled IP receptor to stimulate adenylyl cyclase, raising cAMP levels and leading to activation of protein kinase A (PKA) [12].

In this study, we have investigated the possible contribution of variations in the genes encoding these five GPCRs, to the bleeding tendency of patients diagnosed with type 1 VWD and recruited to the MCMDM-1VWD study. Our results reveal enrichment of rare and novel GPCR gene variations among patients with type 1 VWD, which would support a contribution to the bleeding phenotype.

## Subjects and Methods

### Genetic studies

The exonic and flanking intronic sequences of *P2RY1*, *F2R*, *F2RL3*, *TBXA2R* and *PTGIR* encoding the GPCRs P2Y1, PAR1, PAR4, TP and IP respectively were amplified from the genomic DNA of 146 index cases recruited through the MCMDM-1VWD study and sequenced on an automated ABI 3730 DNA capillary sequencer as described earlier [7]. Where a novel sequence variation was identified in an index case, this was sought in unrelated control subjects recruited through the same centre as the index case. The number of control subjects tested varied between centres though a minimum of 80 subjects were tested in all cases.

The MCMDM-1VWD study was supported by the European Community under the Fifth Framework Program (QLG1-CT-2000-00387) and recruited a total of 154 families historically diagnosed as having type 1 VWD by their treatment centre based on International Society on Thrombosis and Haemostasis-Scientific and Standardization Committee on VWF guidelines. The study protocol was approved by the ethics review committees at the following participating centres: South Sheffield Research Ethics Committee (Sheffield, UK); Informe del Comité Ético de Investigación Clinica de Galicia, Santiago de Compostela (La Coruña, Spain); Comité Consultatif de Protection des Personnes dans la Recherche Biomédicale de Bicêtre, Hôpital Bicêtre (Paris, France); De Commissie Medische Ethiek, Leids Universitair Medisch Centrum (Leiden, The Netherlands); Ethik Kommission der Ärztekammer (Hamburg, Germany); Aarhus University Hospital, Skejby (Aarhus, Denmark); Forskningsetikkommittén I Lund/Malmö, Medicinska Fakulteten, Lunds Universitet (Malmö, Sweden); Leicestershire Research Ethics Committee (Leicester, UK); and South Birmingham Local Research Ethics Committee (Birmingham, UK). In accordance with the Declaration of Helsinki, signed informed consent was obtained from all individuals at the time of recruitment using consent forms in the individual’s own language [3].

### *In silico* analysis of GPCR gene variations

The potential effects of non-synonymous substitutions on protein function were predicted using the online Sorting Intolerant From Tolerant (SIFT; http://sift.bii.a-star.edu.sg/), Polymorphism Phenotyping v2 (PolyPhen-2; http://genetics.bwh.harvard.edu/pph2/) and MutationTaster (http://www.mutationtaster.org/) programs. The potential effects of synonymous variations on splicing were predicted using Human Splice Finder (HSF; http://www.umd.be/HSF/), SplicePort (http://spliceport.cbcb.umd.edu/) and Alternative Splice Site Predictor (ASSP; http://wangcomputing.com/assp/index.html). A multi-species alignment was used to examine conservation of the 5’ untranslated region (UTR) of the *F2R* gene using the Ensembl orthologues alignment tool available at http://www.ensembl.org. The frequencies of usage of different codons were obtained from the online Graphical Codon Usage Analyser tool (http://gcua.schoedl.de/).

### *F2R* reporter gene constructs and luciferase assays

A fragment spanning nucleotides -453 to +50 of the *F2R* gene was amplified from the DNA of an index case who was found to be heterozygous for a single nucleotide transition in the *F2R* 5’UTR (c.-67G>C). The primers were designed to incorporate restriction sites for *Xho* I and *Hind* III at the 5’ and 3’ ends respectively of the amplified product thereby facilitating directional cloning into pGL3.10[luc2] upstream of the firefly luciferase gene to derive wild type -67G and variant -67C *F2R* reporter gene constructs. HEK293T cells [13], maintained in DMEM supplemented with 10% fetal calf serum (FCS) were seeded at a density of 2x10^4^ cells/well in 24-well plates and allowed to adhere for 24 hours. Cells were then transfected with 200 ng of either the -67G or -67C *F2R* luciferase reporter construct, or the empty vector (pGL3.10[luc2]) and 200 ng of the Renilla luciferase reporter, pRLnull, as a control (Promega, UK) using Lipofectamine LTX (Life Technologies, UK) according to manufacturers’ instructions. Forty-eight hours post-transfection, cells were lysed and luciferase activity assessed using the Dual Luciferase Reporter Assay system (Promega, UK). Luciferase levels were normalised for transfection efficiency using Renilla activity and data analysed using an unpaired t-test, with p values less than 0.05 being considered significant.

### *TBXA2R* mini-gene constructs and mini-gene expression analysis

A 1,221 bp fragment corresponding to all of exon 2 and 120 bp of each of the flanking introns of *TBXA2R* was synthesised and supplied in the plasmid pBMH (Biomatik, US). The fragment was removed by *Hind* III digestion and cloned into the pET01 Exontrap cloning vector (MoBiTec, Germany) to derive pET-TBXA2R-WT. The c.654C>T SNV identified in *TBXA2R* was then introduced into pET-TBXA2R-WT by site-directed mutagenesis using the Stratagene QuikChange Site-Directed Mutagenesis Kit according to manufacturers’ instructions, and the orientation and integrity of the cloned WT and variant *TBXA2R* sequences confirmed by sequencing. HEK293 cells were transfected with either the WT or variant *TBXA2R* exontrap vectors using Lipofectamine LTX (Life Technologies, UK). Forty eight hours later, the cells were harvested and RNA isolated using the EZ-RNA Total RNA Isolation Kit (Geneflow, UK). RNA was transcribed to cDNA using a primer specific for the 3’ exon located in the exontrap vector, and the cloned TBXA2R fragments and flanking vector sequences were amplified by PCR using oligonucleotide primers specific for the 5’ and 3’ exons located in the backbone of the exontrap vector.

### PAR1 and PAR4 expression studies

CFP-tagged wild-type (WT) PAR4 and GFP-tagged WT PAR1 expression constructs were generated as previously described [14] and were a kind gift from Professor R Plevin (University of Strathclyde). Mutations were introduced using a Stratagene QuikChange Site-Directed Mutagenesis Kit by polymerase chain reaction amplification. Subsequent products were transformed into DH5a cells, ampicillin-resistant colonies were amplified and the presence of the mutation confirmed by sequencing. HEK293 cells were maintained in DMEM supplemented with 10% FCS, penicillin (100 units/ml) and streptomycin (100 μg/ml). Cells were grown to 50% confluency on coverslips or imaging dishes coated with 0.1mg/mL Poly-D-Lysine (Sigma, UK) and transfected with PAR1 or PAR4 receptor expression plasmids using JET PEI (Polyplus, UK) according to manufacturers’ instructions.

Where expression was quantified, transfection efficiency was calculated by counting the number of DAPI-stained cells that were CFP positive. Mean data was taken from 3 independent experiments.

### Western Blotting

Cells were lysed and solubilised in RIPA lysis buffer [50 mM Tris, 10 mM ethylenediaminetetraacetic acid (EDTA), 150 mM NaCl, 0.5% deoxycholate, 0.1% SDS, 1% Triton-X-100, pH 7.5) containing protease inhibitors (Roche). Samples were separated by SDS-PAGE and subjected to immunoblotting using an anti-GFP antibody (Roche, 1:1000).

### Single-Cell Ca^2+^ Imaging

HEK293 cells were grown on 35 mm glass bottomed Microwell dishes (MatTek, UK) for imaging and transfected with CFP or GFP-tagged WT or mutant PAR receptor DNA. Cells were washed with HEPES-buffered saline buffer (HBBS; 130 mM NaCl, 3 mM KCl, 10 mM HEPES, 1 mM MgCl_2_, 2 mM CaCl_2_ and 30 mM D-glucose, pH 7.3) and loaded with 3μM Fura-2 calcium sensitive dye for 1 hour at 37°C. Cells were washed with HBBS and maintained at room temperature. Ca^2+^ imaging was carried out on a Leica DMIRBE inverted widefield microscope. A single snapshot of cells was captured using a CFP filter prior to live cell imaging to allow identification of transfected cells. Ratiometric fluorescence imaging was performed under control of MetaFluor Imaging software (Molecular Devices). A charge-coupled device camera (CoolSNAP HQ2, Photometrics) was used to collect fluorescence images (emission wavelength, ∼515 nm) from a 40× objective. Pair-wise exposures to 340 and 380 nm of excitation light were provided by a Sutter DG-5 Plus illumination source (Sutter Instrument Company). Drugs were applied directly to the imaging dish by pipette. All experiments were performed at room temperature. Changes in single cell intracellular Ca^2+^ in transfected (GFP or CFP- positive) and non-transfected (GFP or CFP-negative) cells were measured using the CFP fluorescence to identify regions of interest. All data are presented as the change in the 340:380 ratio.

## Results

### Study design and evaluation of bleeding symptoms

The MCMDM-1VWD study aimed to determine the value of clinical, phenotypic and molecular markers for the diagnosis of type 1 VWD by undertaking an extensive assessment of the clinical and laboratory phenotype, and *VWF* analysis in families diagnosed with type 1 VWD. In total, 154 index cases historically diagnosed with type 1 VWD were recruited through 14 participating treatment centres in 9 European countries. Cases were recruited to reflect the full clinical spectrum of type 1 VWD, and therefore included milder cases as well as the more severe highly penetrant type 1 VWD. In addition to the index cases, between 74 and 105 healthy control subjects from each of the participating treatment centres were recruited to the study. None of the control subjects had sought medical attention for a bleeding disorder prior to being enrolled in the study. All subjects provided blood samples for phenotypic and genotypic analysis as described previously [3]. In addition, bleeding symptoms were recorded retrospectively for all index cases, and a subgroup of the control subjects using a questionnaire to derive a quantitative measure of bleeding known as the bleeding score, a value greater than 3 being considered abnormal [15].

### Identification of single nucleotide variations in GPCR genes

Sequence analysis of *P2RY1*, *F2R*, *F2RL3*, *TBXA2R* and *PTGIR* in DNA from 146 of the index cases recruited to the MCMDM-1VWD study identified seven candidate heterozygous single nucleotide variations (SNVs) in eight index cases denoted P1 to P8 (Table 1). The bleeding scores varied widely among these index cases, ranging from a score of 4 in P1 to a score of 20 in P7 (see Table 1). No SNVs were identified in *P2RY1*. *F2R* SNVs were detected in 2 index cases (P1 and P2); a novel single nucleotide transversion in the 5’UTR (c.-67G>C) was identified in P1, and a non-synonymous SNV (c.1063C>T) predicting a p.L355F substitution in the 7^th^ transmembrane domain of PAR1 was detected in P2. The c.1063C>T SNV is listed on dbSNP as rs140912041, and reported to have a minor allele frequency (MAF) of 1.16x10^-4^ (1/8600 alleles tested) among European Americans. Novel *F2RL3* SNVs were identified in three index cases (P3 to P5). P3 was heterozygous for a non-synonymous SNV in *F2RL3* (c.65C>A) which predicted a p.T22N substitution in PAR4 and was co-inherited with a synonymous SNV (c.6680C>T, p.S218 =) in *TBXA2R* which encodes the thromboxane receptor, TP. Synonymous *F2RL3* SNVs were identified in P4 and P5 (c.402C>G, p.A134 =; c.1029G>C p.V343 =). None of these SNVs was present among at least 80 control subjects recruited through the same centres as the cases. A non-synonymous *PTGIR* SNV (c.44T>C, p.V15A) was identified in 3 index cases from 2 centres (P6 to P8). This SNV is listed on dbSNP as a rare polymorphism (rs200213497; MAF 0.002) and also occurred in 2 control subjects (2 alleles of 326 examined) in this study. Since it was predicted to be benign by three online tools, it was not investigated any further. Further studies were undertaken to investigate the potential functional consequences of the SNVs identified in *F2R* and *F2RL3*.

**Table 1:** GPCR gene variations identified in type 1 VWD patients and their predicted effects.

Gene	Protein	SNV	Effect on Protein	SIFT	PolyPhen v2	Mutation Taster	HSF	SplicePort	ASSP	MAF	Bleeding score
*F2R*	PAR1	c.-67 G>C	N/A	-	-	-	-	-	-	Novel	P1: 4
*F2R*	PAR1	c.1063C>T	p.L355F	Tolerated	Probably damaging	Disease causing	-	-	-	1.16 x 10^−4^	P2: 12
*F2RL3*	PAR4	c.65C>A*	p.T22N	Tolerated	Possibly damaging	Polymorphism	-	-	-	Novel	P3: 17
*F2RL3*	PAR4	c.402C>G	p.A134 =	-	-	Disease causing	No changes	Increased acceptor & donor sites	No changes	0.002	P4: 7
*F2RL3*	PAR4	c.1029G>C	p.V343 =	-	-	Disease causing	No changes	No changes	No changes	Novel	P5: 5
*TBXA2R*	TP	c.654C>T*	p.S218 =	-	-	Disease causing	New donor site	New donor site	Increased donor site	Novel	P3: 17
*PTGIR*	IP	c.43T>C	p.V15A	Tolerated	Benign	Polymorphism	Donor site broken	No changes	No changes	0.002	P6: 7; P7: 20; P8: 6

SNV = Single nucleotide variation; MAF = Minor Allele Frequency;

* co-inherited SNVs identified in index case P3.

### c.-67G>C in the 5’UTR of *F2R* alters transcriptional activity

**Fig 1:**
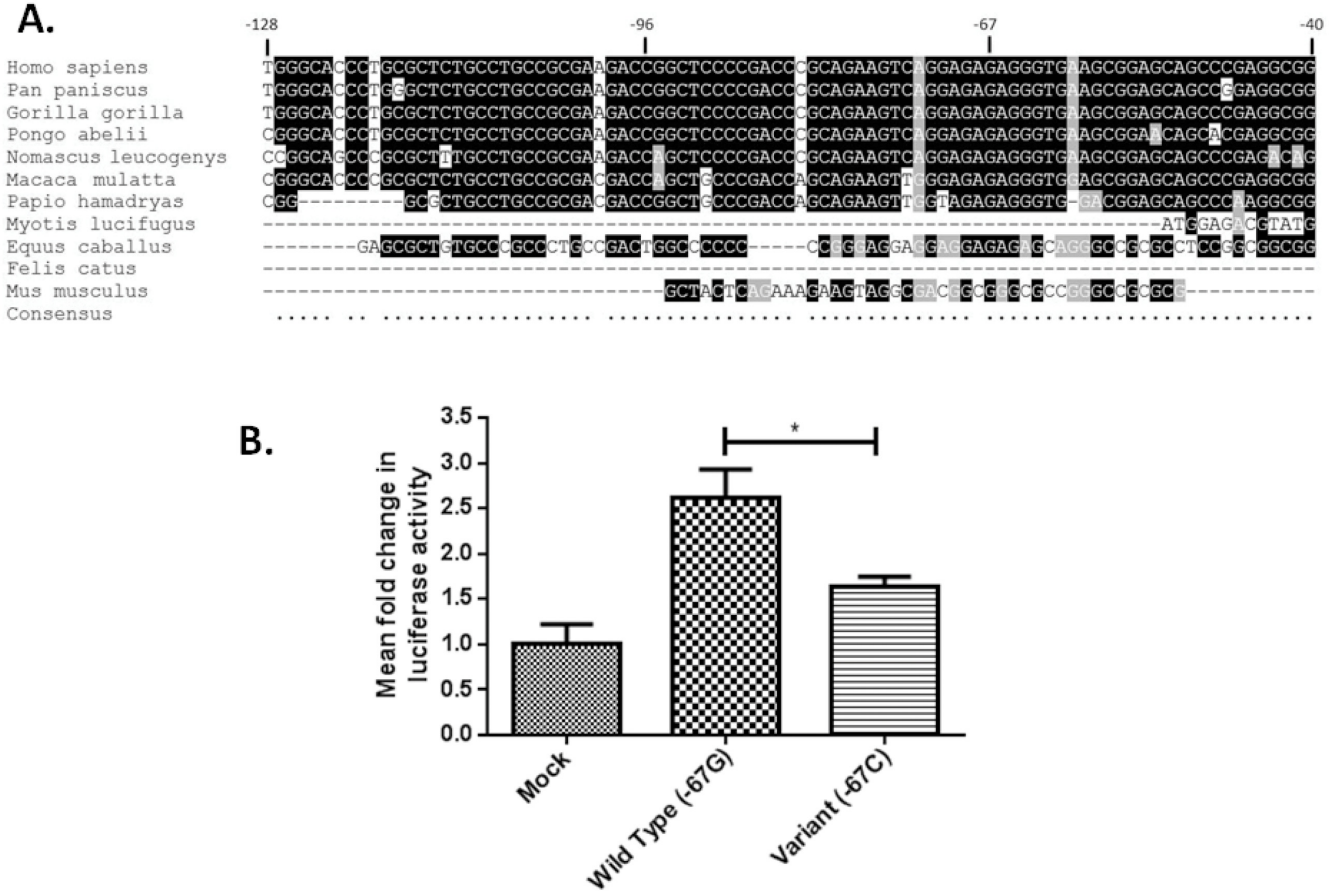
c.-67G>C in the 5’UTR of *F2R* alters transcriptional activity. (A) Alignment of *F2R* 5’UTR nucleotide sequences across multiple species, highly conserved nucleotides are highlighted in black. (B) HEK293T cells were transfected with 200 ng of either -67G or -67C *F2R* luciferase reporter construct, or an empty vector control. Forty-eight hours post-transfection, cells were lysed and luciferase activity assessed. Luciferase levels were normalised for transfection efficiency using Renilla activity. Data shown represents three independent experiments with a minimum of 3 technical replicates to calculate mean fold change ± SE. (*p<0.05).

The *F2R* c.-67G>C transversion identified in P1, occurs in an untranslated region of exon 1 of *F2R*. Comparison of the human *F2R* sequence across this region with the orthologous sequence from 10 other mammalian species revealed that nucleotide -67 was conserved in all 8 of the species having orthologous sequence across the region examined, leading us to speculate that this transversion could influence *F2R* transcription (Fig 1A). This was assessed by transfection of HEK293T cells with luciferase reporter constructs containing 5’UTR sequences of *F2R* (-453 to +50) corresponding to either the -67G (WT) or -67C (variant) allele and determination of the transcriptional activity of these fragments by luciferase assay. The -67G *F2R* reporter construct was shown to have 2.6-fold higher transcriptional activity than the empty vector indicating a potential contribution from the 5’ UTR fragment to *F2R* transcription (Fig 1B). More importantly, the mutated -67C *F2R* construct showed reduced transcriptional activity when compared to the WT -67G construct (p = 0.017) to approximately 1.6-fold that of the empty vector, supporting our prediction that the c.-67G>C SNV affects the transcriptional activity of the *F2R* 5’UTR (Fig 1B).

### p.L355F substitution in PAR1 does not alter PAR1 expression or signalling

**Fig 2:**
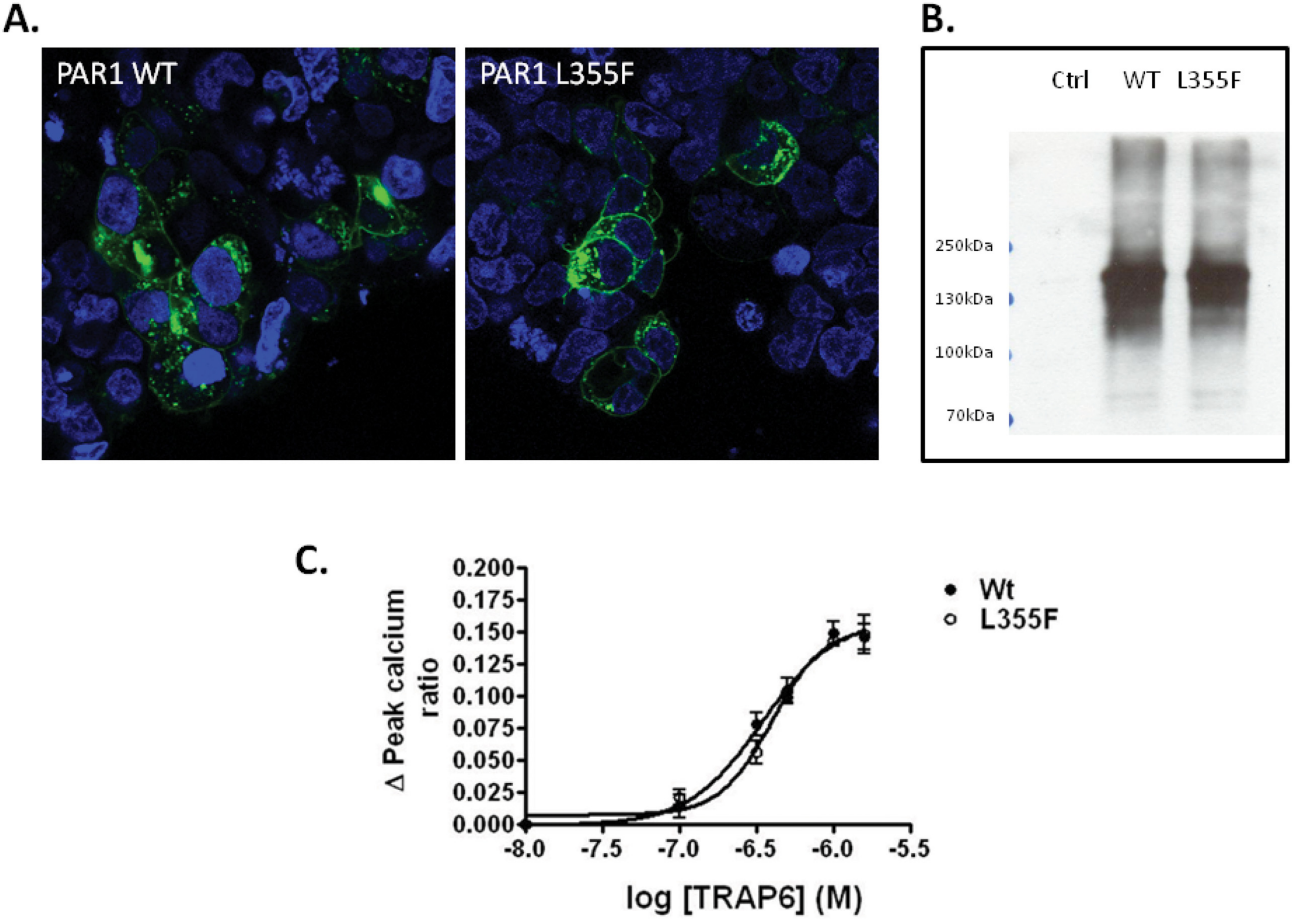
Expression and functional analysis of WT and L355F PAR1 in HEK293 cells. Cells were transfected with WT or L355F GFP-tagged PAR1. (A) Total GFP receptor expression was evaluated by immunofluorescence (shown in green). Cells were also stained with DAPI (shown in blue) to allow comparison of transfection efficiency. Images are representative of 3 independent experiments. (B) Total expression was evaluated by SDS-PAGE of whole cell lysates transfected with WT or L355F GFP-tagged PAR1 and immunoblotting with an anti-GFP antibody. (C) Signalling was assessed by measuring changes in intracellular calcium concentration in response to the PAR1 peptide, TRAP6. Data represents mean±SEM, n = 3.

Leucine 355, which is predicted to be substituted by phenylalanine as a result of the c.1063C>T transition identified in P2, is located in the 7^th^ transmembrane domain of PAR1. This residue is highly conserved across mammalian species and its substitution by phenylalanine was predicted to be “tolerated”, “probably damaging” or “disease causing” by three online tools used to predict the effects of non-synonymous substitutions on protein function (Table 1). We examined the potential effects of the p.L355F substitution on PAR1 signalling capacity in HEK293 cells by expressing GFP-tagged WT and variant L355F PAR1 receptors (Fig 2). The WT and L355F receptors were expressed at similar levels on the surface of HEK cells as assessed by immunofluorescence and western blotting, indicating that the mutation did not disrupt receptor synthesis or trafficking to the membrane (Fig 2A and 2B). Furthermore, the signalling capacity of the variant L355F receptor, as assessed by its ability to direct mobilization of Ca^2+^, was similar to the WT receptor in response to the PAR1 agonist TRAP-6 (Fig 2C). Subsequent assessment of platelet function in the patient using a Multiplate analyser (Roche) revealed normal aggregation to ADP, collagen, TRAP and arachidonic acid suggesting that the p.L355F substitution does not impact on platelet function (data not shown).

### Characterisation of co-inherited *F2RL3* and *TBXA2R* SNVs

**Fig 3:**
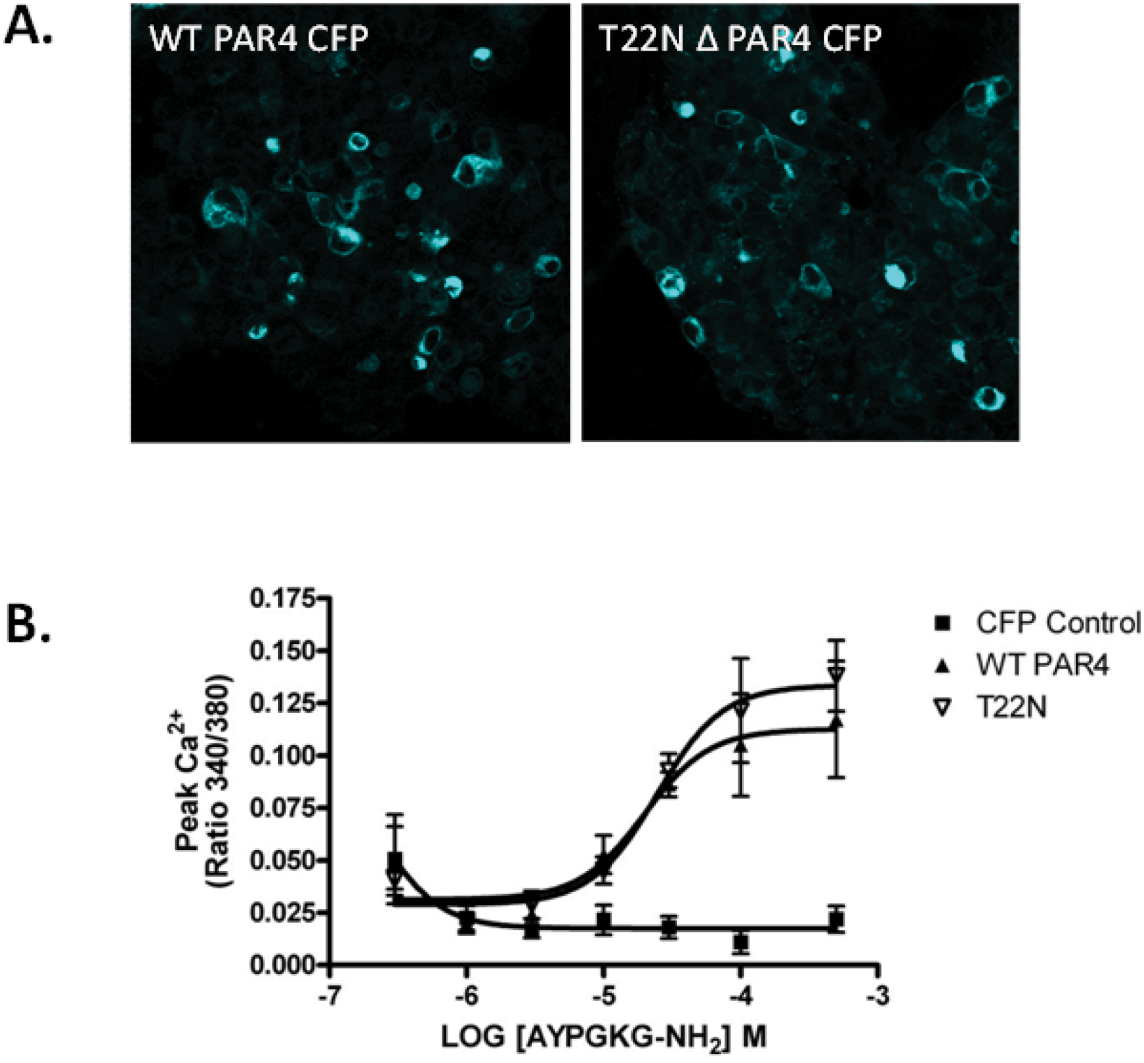
Expression and functional analysis of WT and T22N PAR4 in HEK293 cells. Cells were transfected with WT or T22N CFP-tagged PAR4. (A) Total CFP receptor expression was evaluated by immunofluorescence (shown in blue). Images are representative of 3 independent experiments. (C) Signalling was assessed by measuring changes in intracellular calcium concentration in response to the PAR4 peptide, AYPGKG. Data represents mean±SEM, n = 3.

The p.T22N substitution predicted by the novel c.65C>A *F2RL3* SNV identified in P3 occurs in the N-terminal exodomain of PAR4, in the region of the receptor that is removed by thrombin cleavage to expose the tethered ligand that effects receptor signalling [16]. This substitution was predicted to be “possibly damaging” by one online prediction tool, and tolerated by two other tools (Table 1). We investigated whether the substitution could affect trafficking of PAR4, and thrombin signalling, by expressing CFP-tagged WT and variant T22N PAR4 receptors, which demonstrated that the PAR4 variant was expressed normally on HEK cells and showed no detectable differences in expression or signalling in response to thrombin compared to the WT receptor (Fig 3). The synonymous *TBXA2R* SNV that was also inherited by P3 (c.6680 C>T, p.S218 =) was predicted to be “disease causing” by Mutation Taster, and the three splicing prediction tools HSF, SplicePort and ASSP, predicted the introduction of a cryptic donor splice site in exon 2 of the *TBXA2R* RNA. As P3 was unavailable for further investigation, it was not possible to confirm the predicted effects of the c.6680C>T SNV on *TBXA2R* splicing by platelet RNA analysis. We therefore investigated whether the SNV could alter splicing of the *TBXA2R* RNA by cloning fragments corresponding to either the WT or variant *TBXA2R* sequence into the multiple cloning site of an exon trap vector which contained 5’ and 3’ exons separated by a 600 bp intron sequence into which the *TBXA2R* sequence was cloned. The WT and variant Exontrap vectors were transfected into HEK293 cells, and the RNA resulting from transcription of the vector sequences transcribed to cDNA and then amplified using primers located either in the exonic sequences flanking the inserted *TBXA2R* fragment or within the *TBXA2R* fragment. Repeated sequence analysis of the DNA products revealed no consistent differences in the splicing of the WT and variant constructs (n = 4; data not shown).

### Synonymous *F2RL3* SNVs

**Fig 4:**
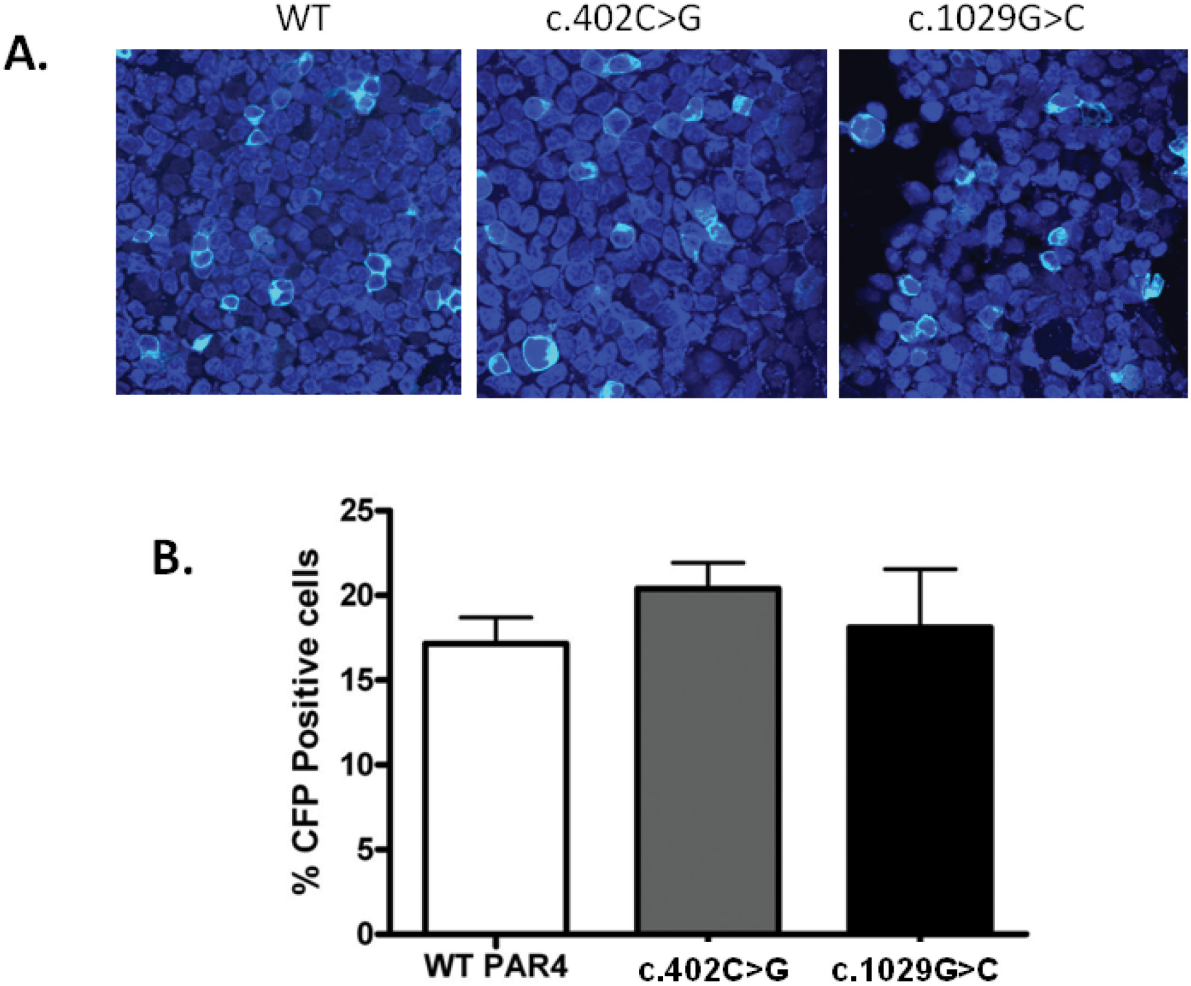
Expression of WT, c.402 and c.1029 PAR4 in HEK293 cells. Cells were transfected with WT or mutant CFP-tagged PAR4. (A) Total CFP receptor expression was evaluated by immunofluorescence (shown in pale blue). Cells were also stained with DAPI (shown in dark blue) to allow comparison of transfection efficiency. Images are representative of 3 independent experiments. (B) Transfection efficiency was quantified by calculating percentage of DAPI-stained cells that were CFP positive. Data represents mean±SEM, n = 3.

The synonymous SNVs affecting codons 134 (c.402C>G; GC**C**>GC**G**; Ala) and 343 (c.1029G>C; GT**G**>GT**C**; Val) in *F2RL3* which were identified in P4 and P5 respectively were both predicted to be “disease causing” by Mutation Taster. One of the splicing prediction tools predicted marginal changes in splicing in the case of the c.402C>G variation, which were not investigated further. Both SNVs resulted in substitutions of codons that were preferentially used in the human genome by less frequently used codons. In the case of the c.402C>G SNV, the most commonly used codon for Ala (GCC; 40%) was substituted by the least used codon (GCG; 11%), while in the case of the c.1029G>C SNV the variation led to substitution of the most commonly used codon for Val (GTG; 46%) by the next most frequent codon (GTC; 24%). Given the increasingly recognised contribution of codon usage bias to differential expression of synonymous alleles, we examined whether the c.402C>G and c.1029G>C SNVs resulted in differential PAR4 gene expression by introducing the SNVs into the CFP-tagged expression construct and expressing the constructs in HEK293 cells. Both variants were expressed at similar levels to the WT PAR4 and there was no difference in the percentage of CFP-positive cells between those cells transfected with the WT construct and those expressing the synonymous variants (Fig 4).

## Discussion

We have identified seven heterozygous SNVs affecting *F2R*, *F2RL3*, *TBXA2R* and *PTGIR* in 8 of 146 patients with a historical diagnosis of type 1 VWD who were enrolled in the MCMDM-1VWD study. Four of the SNVs were previously unreported and were not observed among at least 80 control subjects (160 alleles) recruited to the study through the same centres as the patients. The remaining 3 SNVs (c.1063C>T in *F2R*; C.402C>G in *F2RL3*; c.43T>C in *PTGIR*) were listed on dbSNP as having allele frequencies of 0.002 or less.

To our knowledge, the c.-67G>C SNV identified in P1 is the first *F2R* variant to be identified in an individual with a bleeding disorder. The high conservation of the *F2R* 5’UTR in the region encompassing nucleotide -67 suggested a possible role for this region in regulating *F2R* expression that could be disrupted by the SNV, which we investigated by examining the promoter activity of 5’UTR fragments corresponding to the -67G and -67C alleles in a reporter gene assay. The WT construct showed an increase in luciferase activity, when compared to the empty vector, suggesting a role for the 5’UTR in regulating *F2R* expression, and the reduction in luciferase activity that was observed with the -67C construct suggests that the -67G>C SNV alters transcription factor binding in the 5’UTR. Further investigation will be required to determine the identity of the transcription factor(s) that bind to the 5’UTR to regulate *F2R* expression and mediate the differential transcription caused by the -67G>C transversion. Analysis of the sequence in the region of nucleotide -67 led us to postulate that the glucocorticoid receptor might transactivate the *F2R* gene by binding to a glucocorticoid response element in the region. However, preliminary experiments not reported here in which we co-expressed the -67 reporter plasmids in the presence of glucocorticoid receptor and dexamethasone allowed us to rule out this possibility. Platelet PAR1 receptor levels have previously been shown to be stable over time and to vary 2 to 4-fold between individuals [17,18]. Differences in levels have been reported to be due at least partly to an intronic polymorphism located upstream of exon 2 (rs168753), which also governs platelet response to the PAR1 specific peptide agonist SFLLRN [17]. It is possible that, in the presence of other genetic variations (such as the *VWF* mutation, blood group O), a reduction in *F2R* transcription could also contribute to the bleeding tendency. Indeed, differential expression of *F2R* as a result of polymorphic variation could, in principle, contribute to the variable heritability of type 1 VWD, and may also contribute to the increased risk of bleeding associated with the PAR1 antagonists [19].

The heterozygous p.L355F substitution that was identified in P2 occurred in the 7^th^ transmembrane domain of PAR1, a region encompassing amino acids alanine 349 to tyrosine 371. Crystallographic analysis of this region of the receptor has shown it to be involved in binding to the PAR1 antagonist vorapaxar, which binds to PAR1 to inhibit receptor activation by its tethered ligand [20]. In particular, tyrosine 353 located in this domain hydrogen bonds with tyrosine 183 in the 3^rd^ transmembrane domain to form the base of a binding pocket for vorapaxar. It also forms a hydrogen bond with histidine 255 in the second extracellular loop (ECL2) which contributes to ECL2 having a closed conformation over the ligand-binding pocket. Substitution of tyrosine 353 by alanine was found to lead to a reduction in cell surface expression of the receptor and an associated reduction in agonist peptide activation [20]. In contrast, we showed that substitution of leucine 355 by phenylalanine did not affect expression of PAR1 or its ability to direct calcium mobilization. The p.L355F substitution is listed on dbSNP and population frequency data record it as being present on 1 of 8600 European American and 1 of 4406 African American alleles, suggesting that it may be a rare sequence variant. Similarly, substitution of the adjacent leucine residue by phenylalanine (p.L354F) has also been detected on 1 of 4406 African American alleles, which suggests that substitutions at these positions by nonpolar residues may be tolerated in the receptor, and indeed platelet function as assessed in P1 would appear to support this, though we cannot exclude the possibility that these substitutions have subtle effects on platelet function that may, in combination with other predisposing factors such as a VWF gene defect and blood group O, contribute to a bleeding tendency.

We identified three SNVs in *F2RL3* encoding PAR4, two of which were synonymous changes in the codons for alanine 134 and valine 343 and predicted to be deleterious. Both changes caused alterations from preferentially used to rare codons, leading us to hypothesise that the SNVs could alter the rate of translation or co-translational folding of PAR4 leading to a reduction in expression of the receptor. Similar mechanisms have been proposed to explain changes in substrate specificity in the *MDR1* gene as a result of a synonymous polymorphism, and a case of haemophilia B in which the only underlying genetic defect that could be identified was a synonymous mutation [21,22]. However, *in vitro* expression of PAR4 constructs bearing the synonymous variations revealed no difference in the levels of expression when compared to the WT constructs, suggesting normal translation and folding of the receptor.

The third, non-synonymous, SNV identified in *F2RL3* predicted a p.T22N substitution in PAR4 and was co-inherited with a synonymous *TBXA2R* SNV that was predicted to alter splicing of the *TBXA2R* RNA. The former was predicted to be tolerated and indeed no difference in expression or signalling capacity of the T22N PAR4 variant was observed compared to the WT receptor after expression in HEK cells. In contrast, the synonymous *TBXA2R* variant was predicted to introduce a new donor splice site which if used, would reduce the length of exon 2 by 134 bp. Splicing of the shorter exon 2 to exon 3, would result in a frameshift and introduction of a premature stop codon 117 codons downstream of codon 218. Unfortunately, as the patient was unavailable for further study, we were unable to confirm this prediction by examination of platelet RNA and instead we investigated whether the c.654C>T *TBXA2R* SNV affected RNA splicing by expressing mini-gene constructs corresponding to the WT and variant alleles of the SNV. The results of these experiments supported the predictions that the variant allele caused differential RNA splicing. However, we have not pursued this approach as the findings were not consistent or reproducible between experiments. Furthermore, while this approach can be helpful in providing some indication as to whether a genetic variation causes differential splicing, the results of the analysis may not accurately reflect *in vivo* splicing of the *TBXA2R* RNA. If the mutation does affect RNA splicing we would predict a partial reduction in surface expression of the thromboxane receptor on platelets and compromised signalling, which could contribute to the bleeding tendency in the patient. To date, five *TBXA2R* defects have been described, four predicting amino acid substitutions (p.R60L, p. D304N, p.W29C, p.N42S) and a fifth frameshift defect caused by a single nucleotide insertion (c.167dupG) [23–27]. All five defects have been found to cause platelet dysfunction when tested *ex vivo*, and all were identified in subjects with mild mucocutaneous bleeding symptoms who were heterozygous for the defects. However, in all cases, asymptomatic relatives who were heterozygous for the same defects were also identified, an observation that has led our group, and others, to conclude that heterozygosity for *TBXA2R* defects may not be sufficient to cause abnormal mucocutaneous bleeding symptoms and indeed it is more likely to be expressed phenotypically when combined with an additional haemostatic defect such as type 1 VWD.

A shortcoming of this study was the lack of availability of index cases for follow up *ex vivo* assessment of platelet function including RNA and flow cytometric analysis, since this would have allowed confirmation of the *in silico* predictions of the effects of SNVs identified in the GPCR genes directly in the patients. We therefore explored the potential effects of the GPCR SNVs identified in the study using heterologous expression systems. These studies provided evidence supporting a functional effect for the c.-67G>C SNV in *F2R* which was found to be associated with differential *F2R* promoter activity in a luciferase reporter assay, though whether this variant is of any significance *in vivo* remains to be seen. In the case of the c.654C>T SNV in *TBXA2R*, while the *in silico* analysis predicted that this would lead to aberrant splicing of the *TBXA2R* RNA, the absence of further samples from the patient precluded platelet RNA analysis to confirm this. On the other hand, heterologous expression studies revealed that the p.L355F PAR1 and p.T22N PAR4 variants, and the two synonymous PAR4 variants (p. A134 = and p.V343 =) all behaved similarly to the counterpart wild-type receptors. Our findings suggest that these variants are therefore unlikely to be of any significance *in vivo*, though we cannot rule out the possibility that they may have subtle effects on receptor expression or function which are not easily detected after overexpression in heterologous cells. Examination of the bleeding scores in the eight index cases with GPCR gene SNVs revealed significant bleeding in all cases, and scores that varied widely from 4 to 20. The bleeding scores in the index cases reflect the experience of bleeding symptoms at the time of historical diagnosis, and increase with age, and the number of haemostatic challenges faced [15]. Given these factors, and that most of the SNVs were identified in single cases, the extent to which any given GPCR SNV contributed to the bleeding tendency could not be determined. Importantly, the lack of correlation between the predicted effects of SNVs made using the bioinformatic tools and the findings of the *in vitro* expression studies, emphasise the importance of using several computational tools to predict the potential effects of candidate gene variants, and of confirming the predictions where possible in appropriate experimental systems [28].

If we include the two *P2RY12* SNVs previously identified in the MCMDM-1VWD cohort, 10 of the 146 index cases studied are heterozygous for GPCR gene SNVs (6.8.%). The enrichment of novel or rare SNVs in GPCR genes mediating platelet aggregation among type 1 VWD patients, and the absence of the same variants from control subjects supports a contribution from loci other than *VWF* to the bleeding phenotype of patients with type 1 VWD, which is consistent with the view of mild type 1 VWD as a polygenic disorder. We anticipate that the application of next generation sequencing technologies to the genetic investigation of patients with type 1 VWD and platelet bleeding disorders will reveal additional cases of these and indeed other rare GPCR variants, which will ultimately allow assessment of their contribution to the bleeding tendency in affected patients.
